# Supplementation of Energy Drinks with Green Tea Extract: Effect on In Vitro Abrasive/Erosive Dentin Wear

**DOI:** 10.3290/j.ohpd.b4586835

**Published:** 2023-11-02

**Authors:** Nicolai Blatter, Blend Hamza, Thomas Attin, Florian J Wegehaupt

**Affiliations:** a Dental Master’s Student, Clinic of Conservative and Preventive Dentistry, Center of Dental Medicine, University of Zürich, Zürich, Switzerland. Performed the experiment in partial fulfilment for master’s degree, wrote the manuscript.; b Senior Teaching and Research Assistant, Clinic of Orthodontics and Pediatric Dentistry, Center of Dental Medicine, University of Zürich, Zürich, Switzerland. Conceived and designed the experiment, critical evaluation of the manuscript, supervision.; c Professor and Clinic Director, Clinic of Conservative and Preventive Dentistry, Center of Dental Medicine, University of Zürich, Zürich, Switzerland. Performed critical evaluation of the manuscript.; d Professor and Head of the Department of Preventive Dentistry and Oral Epidemiology, Center of Dental Medicine, University of Zürich, Zürich, Switzerland. Conceived and designed the experiment, critical evaluation of the manuscript, supervision.

**Keywords:** abrasive dentin wear, energy drinks, erosive dentin wear, green tea, profilometry

## Abstract

**Purpose:**

To investigate the effect of the supplementation of energy drinks with green tea extract on abrasive and erosive dentin wear.

**Materials and Methods:**

Six groups, each comprising 15 bovine dentin samples, were prepared, yielding a total of 90 samples. Erosion was performed by immersing the samples in Red Bull and Red Bull light with and without green tea extract. Tap water with and without green tea extract was used as the control groups. The samples were subjected to abrasive/erosive cycling for five days. The following cycling was performed daily: toothbrush abrasion (20 brushstrokes; 2.5 N); eight erosive cycles (2 min storage in the respective solutions); in between the erosive cycles, storage in artificial saliva (60 min) and again toothbrush abrasion (20 brushstrokes; 2.5 N). During the night, samples were again stored in artificial saliva. Abrasive/erosive dentin wear was measured using a stylus profilometer (µm, accuracy = 40 nm). The measured dentin loss results from the vertical position shift on the y-axis from base to final profile after the wear process in 2D. Pairwise comparisons between the groups were carried out using Wilcoxon signed-rank test.

**Results:**

The following dentin wear (median [IQR]) was measured: Red Bull: 1.9 µm (0.5); Red Bull Light: 1.3 µm (0.3); Red Bull with green tea extract: 0.8 µm (0.3); Red Bull Light with green tea extract: 0.3 µm (0.5); Tap water with green tea extract: -0.2 µm (0.7); Tap water: -1.0 µm (1.2). The comparison of all tested groups to each other proved to be statistically significant (p < 0.05).

**Conclusion:**

The supplementation of energy drinks with green tea extract provide a protective effect against erosive/abrasive wear in vitro.

Erosive tooth wear has increasingly become a considerable problem in dentistry. It is caused by acid attacks that soften and dissolve tooth minerals in the absence of bacteria.^3,6,17,18^ Erosive tooth wear can occur in two different ways: either from intrinsic acid attack (regurgitated gastric acid) or from extrinsically supplied foods and beverages containing acidic components (e.g., soft drinks).^18,21^ Abrasion is another process that leads to tooth wear. It takes place during the mechanical interaction between tooth hard tissue and other materials (mainly abrasives in toothpastes). Studies have shown that both enamel and dentin are more susceptible to increased substance loss by abrasion if first exposed to acid attacks.^21^ Furthermore, it was reported that waiting for the tooth to remineralise (up to 1 h) after consuming acidic beverages, and before subjecting it to abrasion (toothbrushing), did not make it less susceptible to abrasive wear in comparison to no remineralisation time.^2,4^ This actually means that both processes (erosive and abrasive tooth wear) cannot be regarded as fully separate phenomena and clinically go hand in hand.

In order to reduce the problem of erosion to some extent, certain substances have been added to acidic beverages in several studies to reduce their erosive potential, such as green tea extract and calcium.^6,14^ The effect of calcium in reducing the erosive potential was attributed to the fact that an increased calcium content simultaneously results in an increased degree of saturation with respect to tooth minerals of the erosive agent. Thus, less calcium will be chelated from the tooth to reach a chemical equilibrium. Simultaneously, the presence of calcium in the erosive agent increases its pH, which also contributes to the reduced erosivity of the agent. For example, Larsen et al^16^ reported a statistically significant reduction in the erosive tooth wear when the erosive agent (orange juice) was supplemented with high levels of calcium and phosphate. Similar results for calcium supplementation were also reported by Wegehaupt et al.^24^ In a previous study, the addition of 1.2% green tea extract to energy drinks was shown to statistically significantly reduce their erosive potential.^11^ This protective effect against erosive tooth wear has been credited to the presence of epigallocatechin-3-gallate (EGCG), which inhibits matrix metalloproteinases (MMPs).^7^ MMPs are enzymes that can degrade demineralised collagen matrix in dentin. Particular attention was paid to MMPs 2, 8 and 9, which are found in dentin and saliva.^3,7^

As the potential erosion-protective effect of green tea extract has not yet been tested in the presence of an abrasive challenge, this study investigated the effect of supplementing energy drinks with green tea extract on dentin wear under erosive and abrasive conditions. The null hypothesis tested was that the addition of green tea extracts to energy drinks would not reduce the resulting abrasive/erosive dentin wear.

## Materials and Methods

### Preparation of the Samples

Ninety dentin samples were prepared from a total of 15 bovine mandibular anterior teeth. The animals were sacrificed solely for food processing purposes in a local slaughterhouse. Six individual samples were obtained from each anterior tooth. The dentin samples, each with a diameter of 3 mm, were drilled out from the roots of the respective teeth using a trephine diamond bur (Turning & Milling Machine System, PROXXON; Föhren, Germany). Drilling at the roots ensured that the samples only consisted of dentin without enamel. The superficial cementum on the dentin was removed by subsequent grinding. The drilling was carried out under permanent water cooling. In a further step, the obtained samples were embedded in acrylic resin (diameter = 6 mm, height = 3 mm) (Paladur, Heraeus Kulzer; Hanau, Germany) and cured in a polymerisation unit with a setting of 10 min at 45°C and 2 bar (Palamat elite, Heraeus Kulzer). The samples were then ground uniformly on the side to be subjected to erosion and abrasion cycles. Using carborundum paper (SiC Paper, Struers; Ballerup, Denmark), the samples were ground in a two-step grinding programme under constant water cooling inside a grinding machine (Tegramin-30, Struers). In the first pass, each sample was ground with 2000-grit carborundum paper at a contact force of 5 N for 15 s. This carborundum paper was then replaced with a 4000-grit paper. In this second grinding pass, the contact pressure of 5 N was retained, but the grinding time was extended to 30 s. As mentioned above, six samples were obtained from each of the 15 teeth. Each individual sample was then coded (A to F) and randomly assigned to the six experimental groups using an Excel randomising table, taking into account that each experimental group contained only one of the 6 samples taken from each of the 15 teeth. Two parallel lines were engraved into each dentin sample with a custom-made apparatus (in-house production, ZPZ [Clinic for Conservative and Preventive Dentistry], ZZM [Center of Dental Medicine], UZH [University of Zürich], Switzerland). Each sample was fixed inside the apparatus, which was equipped with a sharp tip moving only on a fixed axis. The sharp tip was then put in contact with the sample (like a pen) and a line was engraved along the fixed axis. The engraved lines were made close to dentin but completely in the acrylic resin. Those lines served as orientation for the subsequent profilometric evaluation. Furthermore, some dentin surface was protected from the erosive and abrasive attacks with a piece of adhesive tape (Scotch Crystal Tape 600, 3M; Rüschlikon, Switzerland). The tape was applied in such a way that both the engraved lines and a small part of the dentin were covered (0.5 mm on two sides). Consequently, the actual erosion and abrasion attacks took place only on an approximately 2-mm-wide area of the dentin surface. By re-measuring with a scale, this was kept as consistent as possible across all samples.

### Erosion and Abrasion of the Samples

The samples were allocated into the six experimental groups according to the solutions to be investigated: Zürich tap water (0.07 ppm fluoride, pH = 7.6); 1.2% green tea extract (0.39 ppm fluoride, pH = 5.8, OM24, Omnimedica; Schlieren, Switzerland) with Zürich tap water; Red Bull (pH = 3.5, Red Bull; Baar, Switzerland); Red Bull + green tea extract (pH = 3.7); Red Bull Light (pH = 3.5); Red Bull Light + green tea extract (pH = 3.7).^11^

Each of these groups underwent the following cycles for 5 days:

First, each sample underwent 10 brushing cycles, which equals 20 back-and-forth brush strokes. For this purpose, the samples were placed in a container and covered with a toothpaste slurry made by mixing a toothpaste (Elmex caries protection; Therwil, Switzerland) with artificial saliva at a 1:2 ratio.^15^ Brushing was performed in a custom-built brushing machine (in-house manufacture, ZPZ, ZZM, University of Zürich, Zürich, Switzerland). The brush head (Paro M43, Esro; Thalwil, Switzerland) scrubbed the samples with a pressure of 2.5 N. After this brushing cycle, the samples were rinsed with deionised water and subsequently exposed to 8 cycles of erosive attacks. At the beginning of each erosive cycle, samples were exposed to the respective erosion formula (according to the tested groups) for 2 min at 25°C, by pipetting 8 ml into the side of the sample container by using a ml-graduated pipette (2 samples per container) without agitation. After the erosive attack, the samples were briefly rinsed with deionised water and then stored in artificial saliva for 60 min at 37°C. After the above erosive procedure was repeated 8 times in one day, the samples were brushed under the same condition as at the beginning of the daily cycle. The samples were rinsed again with deionised water and stored in artificial saliva at 37°C for the rest of the day.

### Analysis of Dentin Wear

**Fig 1:**
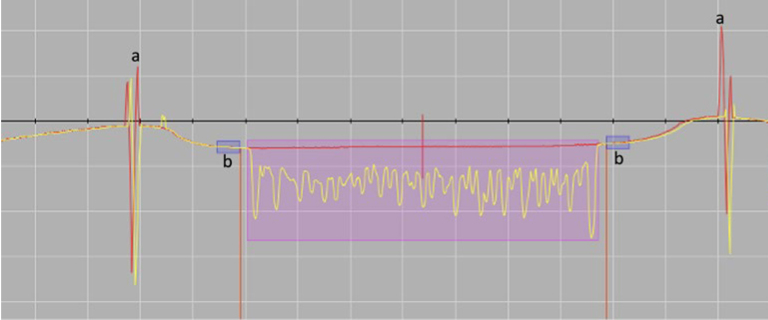
Superimposition of two profiles (red line: baseline profile; yellow line: final profile). The height difference (vertical profile shift on the y-axis) reflects the erosive/abrasive wear and is automatically calculated (in µm) by the software and a mean value is given for each single recorded profile. The letter (a) represents the scratches engraved in the sample. The letter (b) represents the end of the area covered with the adhesive tape.

**Fig 2:**
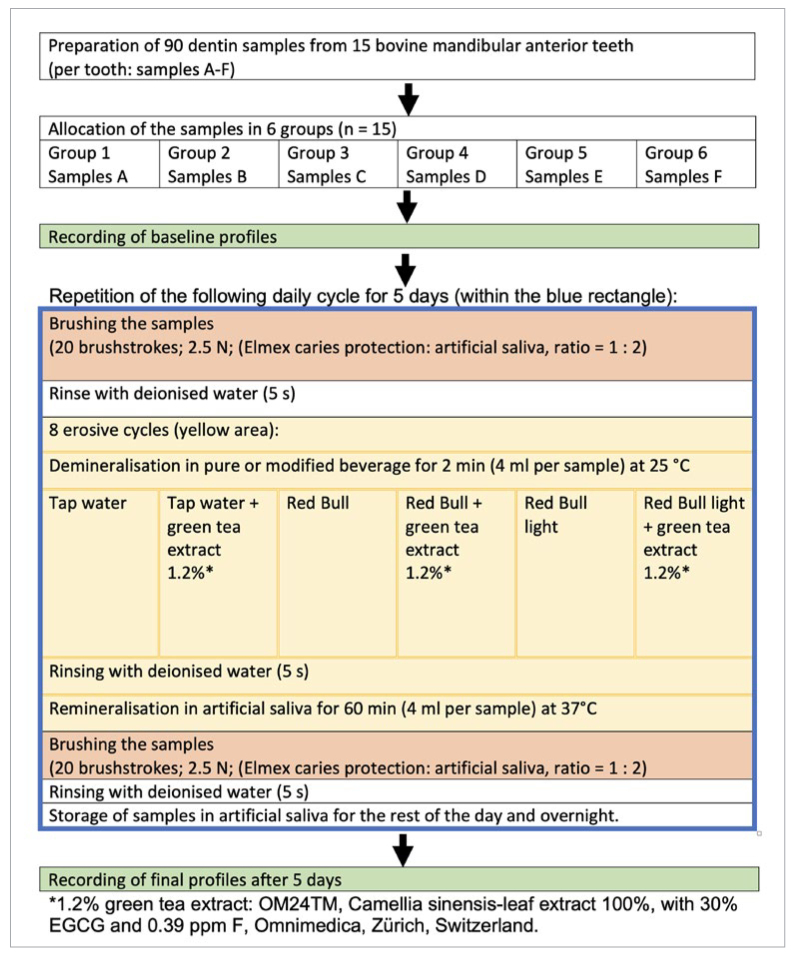
Study design.

Prior to the erosive and abrasive attacks, baseline profiles were recorded using a stylus profilometer (Perthometer S2, Mahr; Göttingen, Germany). After completion of all erosive and abrasive attacks on the dentin samples (i.e., after 5 days), the final profiles were recorded. Five parallel profiles were recorded per surface with a spacing of 250 µm and a measuring distance of 4.8 mm. The exact positioning (at baseline recording) and repositioning (at final profile recording) of each sample in the profilometer was ensured using a prefabricated jig. The accuracy of the profilometric recording was 40 nm. Custom-designed computer software (4D client custom-designed software, ZPZ, ZZM, UZH, Switzerland) stored all measured values. Superimposing the initially recorded baseline profiles on the final profiles showed the vertical change in the dentin surface of each sample (i.e., the resulting erosive and abrasive dentin wear) (Fig 1). The resulting abrasive/erosive dentin wear was presented as positive values, while negative values mean a material gain on the surface of the sample. For exact calculation, the unchanged engraved lines – which were protected by the adhesive tape during the abrasive and erosive cycles – were used. These served as a starting point for an accurate overlay before and after the cycles. This analysis procedure was adopted from a previous study and was undertaken under wet conditions to prevent dentin collagen from desiccating.^1,11^ Figure 2 outlines the study design.

### Statistical Analysis

The Shapiro-Wilk test revealed that normality was not given. Median and interquartile ranges (IQR) of the abrasive and erosive dentin wear in the experimental groups were calculated. Pairwise comparisons between the groups were carried out using the Wilcoxon signed-rank test. The p-value was corrected according to Holm (α = 0.05). All data were analysed using the statistical programme R (The R Foundation for Statistical Computing; Vienna, Austria; www.R-project.org).

## Results

For abrasive and erosive dentin wear, the median and IQR were calculated as follows: tap water: -1.0 µm (1.2); tap water with green tea extract: -0.2 µm (0.7); Red Bull: 1.9 µm (0.5); Red Bull with green tea extract: 0.8 µm (0.3); Red Bull Light: 1.3 µm (0.3); Red Bull Light with green tea extract: 0.3 µm (0.5). The comparison of all tested groups to each other proved to be statistically significant (p < 0.05). Table 1 shows the dentin wear in each group. The most important finding was that the wear of Red Bull without green tea supplement was statistically significantly higher than that of Red Bull with green tea extract (p = 0.0009). Also, the wear of Red Bull Light without green tea was statistically significantly higher than that of Red Bull Light with supplementation (p = 0.0009). Furthermore, a statistically significant difference in the dentin wear between tap water and tap water + green tea extract was found (p = 0.018).

**Table 1:** Abrasive/erosive dentin wear (in µm)

Solutions	Median (IQR)	Mean (± SD)	Minimum	Maximum
Tap water	-0.78 (1.20)A	-1.03 ± 1.08	-3.37	0.93
Tap water with green tea extract	-0.20 (0.65)B	-0.24 ± 0.46	-1.86	0.45
Red Bull	2.04 (0.50)C	1.95 ± 0.36	1.16	2.65
Red Bull with green tea extract	0.80 (0.39)D	0.77 ± 1.08	0.34	1.28
Red Bull Light	1.42 (0.27)E	1.35 ± 0.31	0.95	2.20
Red Bull Light with green tea extract	0.36 (0.50)F	0.31 ± 0.43	-0.85	0.99

Positive values indicate dentin material loss and negative values indicate material gain. Different letters indicate a statistically significant difference between the groups. IQR: interquartile range; SD: standard deviation.

## Discussion

In recent years, the consumption of energy drinks has risen dramatically in Europe.^11^ The problem of erosive tooth wear has therefore become an increasingly important clinical problem. The present study emphasises the fact that the supplementation of energy drinks with green tea extract provides protection against tooth wear under abrasive and erosive conditions. In earlier in-vitro studies, this protective effect was investigated and demonstrated, but without a simulated abrasive challenge in addition to the erosive one.^6,8,11,19^ Therefore, this study was conducted to investigate whether the reported erosion-protective effect would still occur under additional abrasive conditions, which better mimic the clinical situation.

Bovine permanent mandibular anterior teeth were used in this study. Bovine teeth offer many advantages over human teeth. On the one hand, they are more readily available in sufficient quantities. On the other hand, bovine teeth are much larger, which means that several samples can be made from a single tooth. In addition, the teeth normally do not show caries, which means that a more homogeneous mineral content within the tooth structure can be expected.^26^ Furthermore, it has been shown that bovine teeth can be compared to human teeth under erosive as well as abrasive conditions. The reason for this is that the teeth do not differ greatly in either the distribution or mineral content.^23^ The actual wear caused by erosion and abrasion was measured and analysed using a stylus profilometer. This measurement method has been widely used in the literature.^5,20^ However, some drawbacks of this method have been described. For instance, the stylus might penetrate the eroded surface, especially in enamel, leading to an overestimation of the erosion depth. Furthermore, non-contact optical/confocal-based profilometry is becoming more popular and could present a reliable and better alternative to contact profilometry.^20^ The fact that six samples were prepared from each of the 15 bovine teeth and divided among the six groups to be analysed brought further homogeneity to the study. The duration of each erosive cycle in this study was set at 2 min and 25°C, and was repeated 8 times in one day. The intervals between each cycle were exactly 60 min, during which the samples were remineralised with artificial saliva at 37°C. This was intended to reflect the clinical situation of repeatedly – and excessively – consuming a beverage throughout the day. In addition, the abrasive challenge (toothbrushing) took place before the beginning the erosive procedure as well as at the end of all erosive cycles to reflect the situation of brushing the teeth once in the morning and once at night. Erosive cycles were performed with a fairly well-known brand of energy drinks containing citric acid which have and a pH of 3.5.^11^ Dentin samples were subjected to 20 brushing strokes, which exceed the anticipated number of brushing strokes in vivo: a single tooth would be clinically subjected to 10 to 15 strokes.^25^ The brushing pressure used here, 2.5 N, lies within most frequent brushing pressures applied in similar studies (2 to 3 N).^25^ Toothbrushing was carried out with a manual toothbrush and a toothpaste slurry with an RDA of 77.^10^ Using a sonic toothbrush or a toothpaste with a higher RDA might have led to higher abrasive and erosive wear in this study.^12^ It should also be noted that soft drink consumption clinically occurs at lower temperatures. It is known that the temperature has an influence on the erosive potential of soft drinks: Erosive wear is expected to be higher if the beverage has a higher temperature in the oral cavity at the time of consumption.^22^

The supplementation of energy drinks with green tea extract provided a statistically significant reduction of the erosive/abrasive dentin wear in this study. This protective effect can most probably attributed to the presence of EGCG, a polyphenol found in green tea at a concentration of 30%; it has an inhibitory effect on MMPs. As mentioned above, tooth erosion is a chemical process in which minerals are released from the tooth structure and subsequently degraded in the presence of an acidic solution. After such acidic dissolution, a layer of demineralized exposed collagen matrix is found, which is further degraded by collagenases.^13^ More precisely, this chemical process is accomplished by the MMPs, which are found in saliva and dentin.^7^ It is logical that the inhibition of these MMPs would lead to a reduction of erosion and thus a preservation of the tooth structure. Several studies have reported that EGCG has an inhibitory effect on MMPs.^6,11,18^ It is assumed that the molecular secondary structure of the MMPs is no longer the same after they are exposed to EGCG.^13^ Consequently, the degradation of the exposed matrix by the MMPs is inhibited.^9^ The observed protective effect of green tea was observed in both versions of the tested energy drinks (regular and light). Both groups that were brushed with tap water (with and without green tea) showed negative values, which means that a film precipitated onto the surface of the dentin sample. Similar results were also observed by Hamza et al.^11^ However, in that study, the samples subjected to tap water with green tea showed a thicker precipitated film than the samples subjected only to tap water. The opposite was observed in this study and the samples brushed only with tap water showed a thicker precipitated film than the samples brushed with tap water with green tea (expressed by lower negative values; Table 1). The fact the green-tea–extract powder might have played an abrasive role in this study could explain this interesting, controversial finding. In other words, the toothbrush rubbed the precipitated film of tea extract off the dentin surface to some content in this study, while this film was left undisturbed in the study by Hamza et al,^11^ as no toothbrushing took place.

## Conclusions

The supplementation of energy drinks with green tea extract reduces the erosive potential even under additional abrasive conditions, within the limitations of this study.
